# The Role of Endotoxaemia in the development of Renal Disorders Experimental Obstructive Jaundice in Rats

**DOI:** 10.1155/1996/85863

**Published:** 1996

**Authors:** Janusz Dawiskiba

**Affiliations:** First Clinic of Surgery Medical Academy in Wroclaw 2 Poniatowski str. Wroclaw 50-326 Poland

## Abstract

In rats with 2-week obstructive jaundice the sensitivity to endotoxin was studied and the effect of a single dose of endotoxin on histological development in the kidney, liver and spleen was also investigated. We were tested the effect on accumulation and distribution within organs, of fibrinogen labelled with radioactive iodine 125. We showed an increased sensitivity to endotoxin in obstructive jaundice. The cause of death in most rats was acute circulatory failure during the course of endotoxic shock, without clinical features of disseminated intravascular coagulation. In the isotope study, after endotoxin administration there was a specific dynamic increase of fibrinogen accumulation in the kidneys of rats with obstructive jaundice. We proposed, that the cause of the kidney changes during the course of obstructive jaundice could be the local activation of intrarenal coagulation.


*HPB Surgery*, 1996, Vol.10, pp. 7–10
Reprints available directly from the publisher
Photocopying permitted by license only

© 1996 OPA (Overseas Publishers Association)
Amsterdam B.V. Published in The Netherlands
by Harwood Academic Publishers GmbH
Printed in Malaysia

### The Role of Endotoxaemia in the development of Renal Disorders in Experimental Obstructive Jaundice in Rats

JANUSZ DAWISKIBA

First Clinic of Surgery Medical Academy in Wroclaw, Poland

*(Received 28 October 1993)*

In rats with 2-week obstructive jaundice the sensitivity to endotoxin was studied and the effect of a single dose of endotoxin on histological development in the kidney, liver and spleen was also investigated. We were tested the effect on accumulation and distribution within organs, of fibrinogen labelled with radioactive iodine I 125. We showed an increased sensitivity to endotoxin in obstructive jaundice. The cause of death in most rats was acute circulatory failure during the course of endotoxic shock, without clinical features of disseminated intravascular coagulation. In the isotope study, after endotoxin administration there was a specific dynamic increase of fibrinogen accumulation in the kidneys of rats with obstructive jaundice. We proposed, that the cause of the kidney changes during the course of obstructive jaundice could be the local activation of intrarenal coagulation.

KEY WORDS: Bile duct obstruction–extra hepatic renal disorders endotoxaemia

### INTRODUCTION

Many clinical observations point to the frequent occurence of different organ complications in patients with obstructive jaundice[1,2]

Postoperative mortality in long standing and complete obstructive jaundice is between 5–25%[3,4]. The kidneys are particularly susceptible to damage in obstructive jaundice[5,6]. Clinical investigations have shown, that in patients with obstructive jaundice with decreased renal function as well as disordered hemostasis, the mortality rises to 74%[7,8]. The causes of kidney damage in obstructive jaundice may include renal hypoxemia[9], hypovolemia[10,11], toxic activity of bile acids and bilirubin[12], disorders of the coagulating system[7,13,14] as well as metabolic disorders[15]. In light of both clinical and experimental studies the main cause of renal changes can be attributed to the development of general endotoxaemia[16,17,18]. It also seems that the other causes are only direct or indirect consequences[1,14]. Endotoxaemia during the course of obstructive jaundice is produced by an increased resorption of endotoxins from the digestive tract as well as the disordered mechanism of endotoxin inactivation by the reticuloendothelial system of liver and spleen[20,21,22]

The aim of this experimental study was to assess the sensitivity of rats with obstructive jaundice to endotoxin as well as to determine the nature of organ changes, especially the kidney 1 hour after intravenous administration of endotoxin. In the study we also measured the tissue distribution of fibrinogen in rats with obstructive jaundice after administration of endotoxin.

### MATERIAL AND METHODS

41 male rats of the Buffalo strain, 150–200 g weight were used. The rats were maintained in stable conditions with free access to food and water. They were fed

*Correspondence to*: Janusz Dawiskiba M.D. First Clinic of Surgery Medical Academy in Wroclaw, 50–326 Wroclaw, 2 Poniatowski str., Poland a commercial diet. Procedures were performed under light oxygen-ether anaesthesia in clean, but not sterile conditions. The abdominal cavity was opened with a midline incision after desinfecting the skin. The common bile duct (CBD) was located and obstructive jaundice induced by a double ligation with 5/0 silk and transsection of the CBD in the supraduodenal part between the lowermost tributary of the bile duct and the uppermost tributary of the pancreatic duct. The control group rats underwent opening of the abdominal cavity and dissection of the CBD without ligation.

At the end of the experiment, after opening of the abdominal cavity, in all rats blood for bilirubin measurement was taken by direct aortic puncture.

In rats with obstructive jaundice the bilirubin level was high, the liver was markedly enlarged with characteristic discolouration and the CBD was significantly dilated.

In the study we used endotoxin (Lipopoly saccharide E. coli 0111: B4-Sigma Chem.Co.St.Louis, USA, art. No (L-2630) administered intravenously through the tail vein at 1 mg/kg body weight.

In the earlier part of the experiment we assessed survival after endotoxin administration, and in the later part of the study the effect of endotoxin on producing histological changes in parenchymal organs was determined. In both groups of rats specimens of kidney, liver and lung were taken for histology. They were preserved in 8% formaldehyde and stained with hematoxylin and eosin (H-E) as well as the PTAH method for the presence of fibrinogen.

In the isotopic part of the experiment the effect of obstructive jaundice on the accumulation and distribution of fibrinogen was studied. We used I 125 labelled lyophilized human fibrinogen produced by the Isotopic Institute of Izinta–Budapest–Hungary (code I-JC-15).

In both groups on day 13 from the date of surgery, under light ether anaesthesia, 20 uCi of labelled fibrinogen in 0,5 ml of 0,9% NaCl was introduced through the tail vein. 24 hours later the rats were again sedated and the same route was used for the administration of endotoxin, which was prepared each time by dissolving 1 mg endotoxin in 2,5 ml of 0,9% NaCl. Depending on body weight 0,4 – 0,5 ml of endotoxin solution was given.

1 hour later the abdominal cavity was opened and through direct puncture of the aorta 1 ml of blood was taken to study the isotope activity and a further amount for the bilirubin level. Both kidneys, lungs and liver were removed quickly. Fragments of these organs were placed separately in plastic containers. The remaining organs were placed in weighed containers. The radioactivity in 1 ml of blood as well as the organ specimens were measured by scintillation counting for 50 seconds.

The results of both groups were expressed as mean values with standard deviation. The differences were determined using student *t*-test at a significance level of 0,05.

### RESULTS

24-hour survival of the control group of rats as well as the 2-week obstructive jaundice after endotoxin administration is presented in Table 1. All the rats in the control group survived but only 27% of the 2-week jaundiced rats. Most of the rats in this group died in the 3–4 hours after giving endotoxin.

Histology of organs 1 hour after endotoxin administration revealed in the kidneys a significant broadening of renal canaliculi and acute stasis in renal glomeruli and in the lungs features of moderate emphysema. In addition we noted significant blood stasis in the vascular bed of organs. There was no necrosis demonstrated in renal canaliculi nor glomeruli. Despite specific staining in the studied organs there was no demonstrable accumulation of fibrinogen deposits nor intravascular thrombosis.

The relative kidney, liver and lung weight expressed in grammes/100 grammes of body weight is presented in Table 2. In this way the different rats body weights were taken into account. A significant increase of the

**Table 1** 24-hour survival of rats after intravenous administration of endotoxin E.coli 0111:B4 in dose of 1 mg/kg b.w.

| | *Number of rats* | *24-hour survival* | *percentage (%)* |
|---|---|---|---|
| Control group | 9 | 9 | 100,0 |
| 2-week obstructive jaundice | 11 | 3 | 27,0 |

**Table 2** Relative organ weight in grammes/100 g of b.w.

| *Organ* | *Control group of rats No=10 Mean±SD* | *2-week obstructive jaundice No=11 Mean±SD* | *Statistical difference* |
|---|---|---|---|
| Kidney | 0,79±0,04 | 0,95±0,05 | p<0,001 |
| Liver | 4,95±0,27 | 7,32±0,80 | p<0,001 |
| Lung | 0,58±0,05 | 0,62±0,05 | NSD |
| Bilirubin level (µmol/l) | 9,30–3,80 | 121,60–13,60 | p<0,001 |

relative kidney and liver weight in the 2-week obstructive jaundiced rats was noted, however the relative lung weight of both groups remained the same. The high level of bilirubin after 2-week obstructive jaundice confirmed the presence of extrahepatic cholestasis.

In Table 3 is presented the accumulation of labelled fibrinogen in the kidneys, lungs and liver, shown as the ratio of the level of radiation in 1 g of tissue to the activity in 1 ml blood 1 hour after endotoxin administration.

There was a clear increase in fibrinogen accumulation in the rat kidneys after 2-week obstructive jaundice, the differences were statistically significant at the level of 0,001. No difference in accumulation of fibrinogen was noted in the lung and the accumulation in the liver of the jaundiced rats was lower than in the control group.

### DISCUSSION

Many clinical observations and experimental studies concerning extrahepatic cholestasis show the presence of endotoxins in portal blood as well as in peripheral blood[16,17,23]. The primary cause of endotoxaemia in obstructive jaundice is the lack of bile acids in the digestive tract[24].

Many authors have shown the benefit of using oral bile salts in obstructive jaundice[17,25,26]. Overcomming the defensive barrier of the liver in jaundiced patients leads to the development of endotoxaemia as well as various organ changes[7,16,27].

Our experiment shows an increased sensitivity of rats to obstructive jaundice. Many authors confirm this observation[24,28]. In rats with 2-weeks obstructive jaundice intravenous administration of a single dose of endotoxin in this study was in 24-hours 73% compared with the control group, where all rats survived.

**Table 3** Accumulation of fibrinogen labelled iodine I 125 in organs expressed by the relation of radioactivities of 1 g of tissue to 1 ml of blood 1 hour after i.v. administration of endotoxin E.coli 0111:B4 in dose of 1 mg/kg b.w.

| *Organ* | *Control group or rats No=10 Mean±SD* | *2-week obstructive jaundice No=11 Mean±SD* | *Statistical difference* |
|---|---|---|---|
| Kidney | 0,62±0,09 | 0,95±0,13 | p<0,001 |
| Liver | 2,51±0,07 | 0,41±0,05 | p<0,001 |
| Lung | 0,49±0,03 | 0,48±0,07 | NS |

The mechanism of this specific sensitivity to endotoxin in obstructive jaundice has not been definitely explained at present. Fletcher *et al.*[13]. and Hunt *et al*[14]. relate it to general hemostatic disorders with intravascular coagulation, which are halted by prostacyclins and indomethacin. This is also confirmed by the studies of Wardle *et al.*[28], who in experiments in short duration obstructive jaundice of 10 minutes after endotoxin administration observed a general hypercoagulability in the blood, especially after selective blocade of the reticulo-endothelial system of the liver and spleen.

The kidneys are generally regarded as specifically sensitive to endotoxin[2,27]. Chronic endotoxaemia in obstructive jaundice could be similar, in general, to the Schwartzman reaction[28,29]. This is also confirmed by clinical observations as well as autopsy studies of patients with chronic, long standing obstructive jaundice, in whom the kidneys have various pathological changes calculated with deteriorating function[7,817].

In the experiment using radioactively labelled fibrinogen we showed an increased specific accumulation in the kidneys, it was normal or even decreased in other organs. Similar changes in obstructive jaundice were noted by Fletcher *et al.*[13] and Sagar *et al.*[30]. In these studies intravascular coagulation or fibrinogen deposits in kidneys, which does not, however, exclude a subclinical specific intrarenal activation of coagulation leading to deterioration of function. Local changes in the kidneys was not noted during obstructive jaundice may be discreet and impossible to show using classical clinical methods.

These study results seem to confirm the role of endotoxin in the development of kidneys changes in obstructive jaundice. We support the role of earlier surgical treatment for patients with obstructive jaundice, and the restoration of the bile flow to the digestive tract before radical surgical procedures.

### REFERENCES

1. Amstrong C.P., Dixon J.M., Taylor T.V. and Davies G.C. (1984) Surgical experience of deeply jaundiced patients with bile duct obstruction. *British Journal of Surgery*, **71**: 234–238.
2. Pain J.A., Cahill C.J. and Bailey M.E.(1985) Perioperative complications in obstructive jaundice: therapeutic considerations. *British Journal of Surgery*, **72**: 942–945.
3. Blamey S.L., Fearon K.C.H. Gilmour W.H., Osborne D.H. and Carter D. (1983) Prediction of risk in biliary surgery. *British Journal of Surgery*, **70**: 535–538.
4. Greig J.D., Krukowski Z.H. and Matheson N.A. (1988) Surgical morbidity and mortality in one hundred and twenty nine patients with obstructive jaundice. *Brithish Journal of Surgery*, **75**: 216–219.

5. Thompson J.N., Carolan G., Maywers M.J. and Blumgart L.H. (1989) The perioperative changes in glomerular filtration and renal blood flow in patients with obstructive jaundice. *Acta Chirurgica Scandinavica*, **155**: 465–470.
6. Wilkinson S.P., Moodie H., Stamatakis J.D., Kakkar V.V. and Williams R. (1976) Endotoxaemia and renal failure in cirrhosis and obstructive jaundice. *British Journal of Medicine*, **2**: 1415–1418.
7. Allison M.E.M., Prentice C.R.M., Kennedy A.C. and Blumgart L.H. (1979) Renal function and other factors in obstructive jaundice. *British Journal of Surgery*, **66**: 392–397.
8. Thompson J.N., Edwards W.H., Winearls C.G., Blenkhorn J.I., Benjamin J.S. and Blumgart L.H. (1987) Renal impairment following biliary tract surgery. *British Journal of Surgery*, **74**: 843–847.
9. Dawson J.I. (1965) The incidence of postoperative renal failure in obstructive jaundice. *British Journal of Surgery*, **52**: 663–665.
10. Martinez-Rodenas F., Oms L.M., Carulla X., Segura M., Sancho J.J., Piera C., Fernandez-Espina M.R. and Stiges-Sierra A. (1989) Measurement of body water compartments after ligation of common bile duct in the rabbit. *British Journal of Surgery*, **76**: 461–464.
11. Williams R.D., Elliott D.W. and Zollinger R.M. (1960) The effect of hypotension in obstructive jaundice. *Archives of Surgery*, **81**: 334–340.
12. Baum M., Stirling G.A. and Dawson J.I (1969) Further study into obstructive jaundice and ischaemia renal damage. *British Medical Journal*, **2**:229–231.
13. Fletcher S.M., Westwick J. and Kakkar V.V. (1982) Endotoxin, prostaglandins and renal fibrin deposition in obstructive jaundice. *British Journal of Surgery*, **69**: 625–629.
14. Hunt D.R., Allison M.E.M., Prentice C.R.M. and Blumgart L.H. (1982) Endotoxemia, disturbence of coagulation and obstructive jaundice. *American Journal of Surgery*, **144**: 325–329.
15. Ozawa K., Yamada T., Tanaka J., Ukikusa M. and Tobe T. (1979) The mechanism of suppresion of renal function in patients and rabbits with jaundice. Surgery, *Gynecology and Obstetrics*, **149**: 54–60.
16. Bailey M.E. (1976) Endotoxin, bile salts and renal function in obstructive jaundice. *British Journal of Surgery*, **63**: 774–778.
17. Cahill C.J. (1983) Prevention of postoperative renal failure in patients with obstructive jaundice–the role of bile salts. *British Journal of Surgery*, **70**: 590–595.
18. Greve J.W., Gouma D.J., Soeters P.B. and Burman W.A. (1990) Suppresion of cellular immunity in obstructive jaundice is caused by endotoxin: a study with germ-free rats. *Gastroenterology*, **98**: 478–485.
19. Wardle E.N. (1975) Endotoxaemia and the pathogenesis of acute renal failure. *Quarterly Journal of Medicine*, **44**:389–398.
20. Drivas G., James O. and Wardle N. (1976) Study of reticuloendothelial phagocytic capacity in patients with cholestasis. *British Medical Journal*, **1**: 1568–1569.
21. Pain J.A (1987) Reticuloendothelial function in obstructive jaundice. *British Journal of Surgery*, **74**: 1091–1094.
22. Tanaka N., Rydeb S., Berquist L., Christensen P. and Bengmark S. (1985) Reticuloendothelial function in rats with obstructive jaundice. *British Journal of Surgery*, **72**: 946–949.
23. Gouma D.J., Coelho J.C.U., Fisher D.J., Schleger J.F., Li Y.F. and Moody F.G.(1986) Endotoxemia after relief of biliary obstruction by internal and external drainage in rats. *American Journal of Surgery*, **151**: 476–479.
24. Kocsar L.T., Bertok L. and Varteresz V. (1969) Effect of bile acid on the intestinal absorption of endotoxin in rats. *Journal of Bacteriology*, **100**: 220–223.
25. Evans H.J.R., Torrealba V., Hudd C. and Knight M. (1982) The effect of preoperative bile salt administration on postoperative renal function in patients with obstructive jaundice. *British Journal of Surgery*,**69**: 706–708.
26. Pain J.A. and Bailey M.E. (1988) Prevention of endotoxaemia in obstructive with obstructive jaundice-a comparative study of bile salts. *World Journal of Hepatic, Pancreatic and Biliary Surgery*, **1**: 21–27.
27. Cahill C.J., Pain J.A. and Bailey M.E. (1987) Bile salts, endotoxin and renal function in obstructive jaundice. *Surgery, Gynecology and Obstertrices*, **165**: 519–522.
28. Wardle E.N. and Firight N.A. (1970) Endotoxin and acute renal failure associated with obstructive jaundice. *British Medical Journal*, **4**: 472–474.
29. Hjort P.F and Rapaport S.I. (1965) The Shwartzman reaction: pathogenic mechanisms and clinical manifestation. *Annual Review of Medicine*, **16**: 135–168.
30. Sagar S. and Shields R. (1980) Fibrinogen in hepato–renal syndrome: an experimental study. *British Journal of Surgery*,**67**: 562–564.

